# Twenty-ninth Annual Meeting of the American Medical Association

**Published:** 1878-07

**Authors:** 


### AMERICAN MEDICAL ASSOCIATION.

### Twenty-Ninth Annual Meeting—Held in the City of Buffalo, June 4th, 5th, 6th and 7th, 1878.

(*Reported for the* Chicago Medical Journal and Examiner, *by Roswell Park, M. D., of Chicago.*)

### General Session, Tuesday.

The Association was called to order in St. James Hall at 11 a. m., the President, Dr. T. G. Richardson, of New Orleans, in the Chair. The stage was occupied by the other officers, and later by the Canadian delegates. Prayer was offered by the Rev. Dr. Van Bokkelen, of Buffalo. In answer to the call for the ex-Presidents, Drs. Gross, Davis, Toner, and Bowditch appeared upon the stage. Dr. Thos. F. Rochester, of Buffalo, gave the address of welcome in behalf of the committee of arrangements, offering the freedom of the college and hospitals, and the principal public institutions, declaring that the entire profession of the city had resolved itself into a committee of the whole to assist in making the visitors' stay pleasant and profitable.

Various charges against members or local societies were read and referred to the judicial council. A congratulatory cablegram from Dr. Marion Sims, now in Paris, was read. After some minor business the President delivered the annual address. Without attempting to give even a synopsis of his admirable paper, we will state that he laid particular stress upon the following points: He first alluded to the grand cause of adequate and thorough medical education, and the necessity for a more elevated standard, lamenting that, while the sentiment of the association in times past and the measures proposed were incisive enough, the association had no power to carry them out, and they therefore remained simply as mementoes. He briefly alluded to the history of the movement, and for the first time in the public proceedings of this body, so far as the writer knows, was the Chicago Medical College credited with having taken the initiative in this matter. Being convinced that the current of reformation had taken the right direction, he felt that more must first be done to interest the great body of the profession, instead of assuming the aggressive with the medical colleges. It was from the professional public that the colleges derived their material, and it was only by awakening in these the desire for something higher and better, that the desired end could be attained.

He put in a plea for a more thorough organization of local societies and earnestly hoped that the sixty thousand physicians of this country would affiliate themselves with the Association by means of the usual means of representation.

He found much reason to regret that original investigation was not a more marked feature in the advance of scientific work in this country, especially when contrasting work done here with that done abroad, saying that a few such men as Smithson and Hopkins and Boylston and Toner, were not enough to give the universal encouragement which our home science needed. He recommended either or both of two methods to foster this spirit of original research. One was to concentrate the influence of the entire profession upon congress in order that it might take appropriate measures. In connection with this topic, the speaker referred to the magnificent collections of books and specimens and the work done by the government medical service, and thought these might be made the basis for still more work. His other plan was that the annual dues should be doubled, and the money thus turned into the treasury of the Association should be used in stimulating and rewarding really original work. He had reason to think that if the Association should become incorporated, funds would from time to time be committed to it by will or otherwise for such purposes.

The concluding portions of the address were given to a consideration of State Medicine, whose general questions he considered the most important that ever came up for discussion. He alluded to what had recently been done. Nine State boards of health had been established since the Association, in 1875, first made an appeal to the executive officers of different States. He considered the movement a grand crusade in which every one should be glad to join. He understood the objects of State Medicine to be threefold: First, prevention, by official measures, of the spread of disease not strictly limited to the individual; second, the qualification of men not only for the practice of medicine but for that of public hygiene; and, lastly, the enactment and enforcement of such laws as shall secure to all the benefit of services of the best professional experts in questions of a medico-legal character. If this statement were true how vast the field! The public were fearfully ignorant of hygienic laws, and yet Prof. Tyndall has said, "if anything is to be done in the way of really great sanitary improvement, it must be from the people themselves." But "how shall they (the public) hear without a preacher? And how shall they preach except they be sent?"

Even among the rank and file of the profession, the ignorance in these matters was deplorable. Among other remedies the proper education of the children in our schools, by inculcating the elements of a sound physiology and hygiene must stand prominent. He advocated a national council of health with members from every State, and was grateful for what had been recently done by government, in requiring frequent reports from consuls abroad as to the condition of health in their respective localities.

The address was well received by a large audience, and received frequent and merited bursts of applause.

Dr. White, of Buffalo, 1st Vice-President, who occupied the chair during its delivery, moved that a committee, to consist of the president and his four immediate predecessors in office, be appointed to consider its suggestive points and report. This was seconded by Prof. Gross, in a complimentary speech, and unanimously passed.

Dr. Brodie, of Detroit, read the report of the delegation to the Canadian Medical Association, showing perfect accord and sympathy between the two bodies.

Dr. Sayre also made a verbal report of his visit and warm reception as a delegate to the British Medical Association.

The report by Dr. Seguin, the American delegate, of the conclusions arrived at by the International Medical Congress, held at Geneva, was then read by the Secretary. The American delegates were charged "to advocate the adoption of a progressive uniformity of means of observation and record, with the concurrence, if possible of the members of this congress who should be found there, advocating the application of uniformity in this and other departments of science." For physicians and surgeons the principle accepted by the Congress is a gradual international uniformity of nomenclatures, scales, measures, calibers of instruments, of records of private and hospital practice, physiological experiments, medical clinatology, barometry, thermometry, statistics, etc.

In pharmacy the congress has accepted the conclusions voted at St. Petersburg and Brussels, to wit:

1. The adoption of an universal pharmacopœia, in Latin. 2. The metric system for weights and measures; the centigrade for temperatures. 3. A uniform nomenclature. 4. The chemical preparations to be of determined strength, and the pure drugs to be of assayed strength when possible. 5. Simplification of Galenical preparations, and description according to a uniform plan. 6. The other, and especially more powerful preparations to be made uniform. 7. Physicians to be left free and responsible for non-officinal ingredients and doses of magistral preparations.

The pharmacists now planning a new American Pharmacopœia have been notified of the above, and have been warned that they cannot succeed in founding an uniform pharmacopœia without the concourse of physicians. The report concluded by recommending a commission on uniformity in physic, with power to adjoin specialists in accessory arts, to report from the next meeting of the A. M. A. the standards and conclusions arrived at to the next International Congress, which meets at Amsterdam, three months later; and was signed by Drs. Seguin, Sims and Drysdale.

SECTION MEETINGS, TUESDAY AFTERNOON.

SECTION I. *Practical Medicine, Materia Medica, and Physiology.* Dr. A. S. Loomis, of N. Y., chairman. Dr. Shoemaker, of Pa., read a paper on "Ringworm in Public Institutions," which gave a synopsis of his experience with cause and cure of this affection, and its prevalence in public schools. Some interesting experiments, showing the exchangeability between animals and children were related.

Dr. N. S. Davis, of this city, read a paper upon the "Causes of Pulmonary Tuberculosis." He thought climatic influence was overrated, and that the real causes were lack of proper exercise and food, ill-dressing and dampness. This paper excited general discussion. Dr. Glasgow, of St. Louis, presented a rare and interesting specimen of casts of the smaller bronchi, expectorated in the course of a case of fibrinous or croupous bronchitis, with a verbal report of the same. The Doctor was requested to keep the case under observation, and report at the next meeting, with details of progress and treatment.

SECTION II. *Obstetrics and Gynæcology.*—Dr. Jenks, of Detroit, chairman. Dr. Parvin, of Indiana, read an elaborate paper on "Ovotomy," his name for Dr. Thomas' operation of Laparo-elytrotomy, already referred to and described in this journal. While admiring its ingenuity, he thought the cases would be very rare where the operation would be called for. This paper, with subsequent discussion which it elicited, consumed most of the afternoon.

SECTION III. *Surgery and Anatomy.*—Dr. Henry H. Smith, of Philadelphia, chairman. By special arrangement Dr. Howe, of Buffalo, presented a case of a plastic operation where he had transplanted a piece of integument from the forearm to the lid, with success. He thought the size of piece transplanted justified the exhibition of the case. It was about 6x4 cm.

Dr. Gay, of Buffalo, presented reports of two cases of "Excision of the Diaphysis of the Tibia," where he had removed the entire diaphysis, and where new bone had supplied the place of that removed, while both patients were to-day about on their feet as usual.

Dr. Weeks, of Portland, Me., read a paper on "Septicæmia following Resection of Bones," founded upon a case where it followed the resection of an amputation stump. He took the ground that in septicæmia there is diminished coagulability of blood, while in pyæmia it is increased ; that pyæmia never complicates a case until sufficient time for the formation of pus has elapsed, while septicæmia may take its inception within a very few hours after the injury or operation; the latter being caused by some blood changes, but not by the introduction of pus. In discussing the paper Dr. Keller, of Arkansas, said he had found in some such cases that quinine in large doses would act as a sedative and hypnotic when opium would not.

Dr. Henry H. Martin, of Boston, next detailed his method of doing "Tracheotomy without Tubes," by making the incision as is generally done, and then inserting, with a curved needle on either side, a silk or silver suture through a ring of the trachea and the integument, and thus gently stretching the lips of the wound apart, tying the threads behind the neck, and reinforcing them by strips of adhesive plaster appropriately placed to keep the opening patulous. This method required no case of instruments, and was especially suited for emergencies. By this means he had saved two out of five moribund cases operated upon.

Dr. Carpenter, of Pottsville, Pa., in a paper on the "Identity of Hospital Gangrene with Diphtheria," enumerated the following points of resemblance: Their causation, the interchangeableness of their miasm, their pathological features, their double form and course, the fact that, in both, local infection was preliminary to general, the parallel methods of cure—the same remedies being used with success in either disease, and lastly, similar modes of death and the comparative frequency of heart clot in both diseases. He said that he had met with isolated cases of hospital gangrene in the mountains of Pennsylvania.

A paper by Dr. Sayre was read in abstract, and one by Dr. Clay, of Pennsylvania, on "Peri-typhlitic abcess," by title.

Dr. Alfred Post, of New York, read a paper on "Plastic Surgery," giving reports of cases, and Dr. Moore, of Rochester, described a very ingenious new method of operating antiseptically by utilizing the known properties of carbonic acid gas, simply directing a stream of it upon the parts, or immersing them, as it were, in a stratum of the gas.

SECTION IV. *Medical Jurisprudence, Chemistry and Psychology.*—Dr. Kempster, of Wisconsin, Chairman. This section was not organized till the next day.

SECTION V. *State Medicine and Public Hygiene.*—Dr. Cabell, of the University of Virginia, Chairman. Ex-President Bowditch detailed his patient and laborious "Studies of an epidemic of diphtheria" in Vermont in 1877. He described the locality and traced the epidemic with great care. At its conclusion, he moved its reference to a committee of experts. It was finally, after discussion, referred to the committee of publication.

One of the features of the meeting was a paper by Dr. Seguin, of New York, on the "Intervention of physicians in education." It excited great interest, and is worthy of introduction here in full, did space permit it. He started with the thesis which he had formerly insisted upon, that "new social and individual wants demand new solutions of the problem of education, and most of these expected solutions rest with the physician and the physiologist." The physician's labors should begin, 1st, during vacation. He should then attend to cleanliness, drainage, ventilation, lights, etc., see that the books and charts were suited to the range of vision, the desks to the size of the scholars, and the seats to the diversity of shapes, especially among the girls. 2d. Before school begins, he should thoroughly examine every scholar, recording accurately his results, as to anomalies of size, proportion, vision, or other senses, etc., and decide whether any departure from the ordinary curriculum is called for in individual cases. 3d. He alluded to the increasing prevalence of myopia of school origin, and to the predisposition to hemiplegia later in life, which the habit of working one hand or one side of the body more than the other would cause. 4th. The doctor should make a daily round of the school to guard against the introduction of zymotic or contagious disease, regulate the temperature, discover simulated or dissimulated disease, and to note the effect of courses of studies upon children and any intellectual perturbations. 5th. The physician must interfere in education as soon as the effects of overwork are perceived. 6th. He should create and organize the physiological training and sharpening of the senses and of the hand, taste should be developed and manual dexterity encouraged. 7th. All this time he must keep this record of vital powers, and finally add a summary of the students' physiological powers of perception, action and endurance. In a word, the teachers' certificate should tell what the scholar knows, and the physician's what he can learn. The Doctor concluded by saying that the true riches of this country would be its children, if properly educated.

The paper was emphatically endorsed by those present, and Dr. Frank Hamilton was requested to give a summary of the paper and his own experience in the matter at the meeting in general session the next morning.

### Wednesday Morning—General Session.

The Association was called to order by the President at 9:30 sharp. After election of members by invitation, and reading of letters from Dr. Battey, of Georgia, and Dr. Vaughan, of Mississippi, regretting their inability to attend, the reports of the Judicial Council were called for. None of the charges against members were sustained. It was decided that the local societies of Hot Springs and Garland county, Arkansas, on account of their severance from their State society, should lose their recognition by the association. The report was signed by the Secretary of the council, Dr. Benham.

Dr. N. S. Davis made a special report for the council concerning the charges against the Michigan State Society. The charge was an alleged violation of the code of ethics in electing as a delegate, Prof. E. L. Dunster, of Ann Arbor, knowing him to be engaged in aiding and abetting the graduation of students devoted to an exclusive dogma in practice. The reporter said that careful scrutiny failed to show any clause of the code that referred, even remotely, to the practice that constituted the basis of the above charge. The only clause that could be so considered was that referring to consultation at the bedside. To interpret the conduct of Dr. Dunster as having ignored this clause, would be not only to violate all principles of judicial construction, but would establish a precedent in latitudinous construction of the code, more dangerous to the best interests of the profession than all the evils sought to be remedied. While the council deprecated such aiding and abetting in teaching and graduating students in singular and exclusive dogmas as beneath the dignity of right-minded men in a liberal profession, they found no clause in code, by-laws or constitution, under which the charge could be entertained and adjudicated.

During the excited discussion which this special report provoked, the presiding officer displayed the same familiarity with parliamentary rules which marked his administration during the other sessions. Pending further action, Dr. H. H. Smith delivered his address, as Chairman of Section III.

He took for his subject, "Certain Points in the Pathology of Bone, especially Tubercle." Alluding to the old opinion that the skeleton was simply intended as a support, he went on to say that more recently a new function had been assigned it, the interior of bones being now regarded as a focus of origin of the red and white blood corpuscles, while by the same means septic matter was often introduced into the general circulation. He considered the physiology of red and yellow marrow, and mentioned that the coloring matter of yellow marrow, the corpora lutea, of yolks of eggs, and of ordinary fat, was identical. He quoted most recent investigations by various observers. The lymphatic structures of bone were briefly described, and its great vascularity at greater length. He alluded to the fact that nowhere in bone were the vessels separated by more than the 1-130th of an inch. Realizing these facts, one could readily understand the liability to inflammation and inflammatory changes, especially the formation or "deposition" of tubercle. The speaker mentioned several cases in his own experience where severe osteo-myelitis had followed exposure, without injury or any extension of periosteal inflammation, and said that similar results followed oftener than was generally supposed. One origin of septicæmia could thus be traced; and to prevent this very result he urged the trephining of bones when osteo-myelitis had set in, to give free exit to pus. He mentioned illustrative cases, and said that now no *post-mortem* was complete without a careful examination of the bones.

He referred then at greater length to tubercular changes in bone, and insisted that hip-joint disease, Potts' disease, and similar maladies were much more frequently the result of such change than of injury. He laid great stress upon this matter, and the remainder of his address was devoted to elaborating it and combatting the contrary opinion.

With respect to tubercle his conclusions were:

1. That it was intimately connected with the lymphatics and was deposited in them and in the spongy texture of epiphyses and short bones.

2. That when thus deposited it developed just as when in other situations.

3. That it affected the vessels and cancellated structure rather than the ligaments and cartilages.

4. That the destruction of ligament and cartilage was the effect rather than the cause of impaired nutrition.

5. That softening tubercle caused congestion and inflammation of bone-cells about the deposit.

6. That perverted myeloid cell-action was consequent upon such change, and reacted by modifying the formation of blood corpuscles.

7. That so-called scrofulous disease of bone was essentially perverted myeloid cell action.

The address was received with great interest and was one of the features of the meeting.

Following it, Dr. Hamilton read a synopsis of Dr. Seguin's paper, as requested by the Section of State Medicine, and related in a characteristic way some of the experiences of a few New York City physicians who endeavored, but without success, to have one respectable practitioner appointed upon their city Board of Education. From what he said the writer could but conclude that it was the same old story which Chicagoans have so often heard; that prostituted political influence is supreme where it ought not to be tolerated.

At the conclusion of his remarks the Association unanimously adopted the following resolution:

That in the opinion of this Association medical men ought to have a voice in the construction and location of public school buildings; in the question as to the age at which children should be admitted, the hours of study, and the general management of these institutions; and to this end it is believed to be necessary that one or more intelligent physicians should be placed upon Boards of Education, Boards of Trustees, and upon other similar boards having the control of public education and schools.

Dr. E. W. Jenks, of Detroit, chairman of the Section on Obstetrics and Diseases of Women and Children, then delivered an extended and very able address on "The Causes of Sudden Death of Puerperal Women." Among these causes he particularly mentioned:

1. Lesions of the Circulatory System; especially mitral stenosis, endocarditis, fatty heart, arteritis, phlebitis, thrombosis, embolism, etc.
2. Lesions of the Respiratory System.
3. Lesions of the Nervous System; eclampsia, tetanus, apoplexy, shock, etc.
4. Puerperal Septicæmia.
5. Various other causes, as rupture of uterus, emotion, hæmorrhage, traumatism, etc.

The reading of this address also elicited marked attention.

A recess was then taken to permit the delegates to select the Committee on Nominations.

When called to order again, in reference to the matter of deficiency of the code of ethics, already alluded to in this morning's work, the following preamble and resolution, offered by Dr. J. R. Bronson, of Massachusetts, were referred to the members of the Judicial Council as a committee:

*Whereas*, By the report of the Judicial Council, submitted this day, we are informed that the ethical code of this Association is imperfect, in that it does not recognize by its letter a conceded violation of the spirit of our profession in its relation to irregular medicine; therefore,

*Resolved*, That said Council be instructed to submit to this Association at their meeting, for its consideration, an amendment to the code covering this omission.

A resolution offered by Dr. Foster Pratt, of Kalamazoo, Mich., in relation to the legal status of the insane, was referred to the section on medical jurisprudence.

This closed the morning session.

AFTERNOON — SECTION WORK.

SECTION I. The first paper was one by Dr. G. M. Beard, of New York, upon "Practical Points in the Electrical Treatment of Impotence," which was well received and discussed.

Next was an important paper on "The Neuroses of the Pneumogastric and Sympathetic," by Dr. J. J. Caldwell, of Baltimore. It was discussed by Dr. Palmer, who endorsed the theory presented.

A paper on "The Metric System in Medicine," by Dr. Edward Wigglesworth, of Massachusetts, was read. The system, he stated, has been recommended for use throughout the United States by the State medical societies of both New York and Pennsylvania.

A paper by Dr. Bulkley, of New York, "On the Use of the Solid Rubber Bandage in Treatment of Eczema and Ulcers of the Legs," was read by title, the author being absent.

SECTION II. The first paper was by Dr. Reamy, of Ohio, entitled, "Hour-glass Contraction of the Uterus, Prior to Expulsion of Child, with Case." This was a report of such a case, with inferences and practical deductions. In discussing it Dr. Dunster said he had once seen such a case, where he could see underneath the abdominal walls the longitudinal fibers, only, contracting; he explained such phenomena on the ground of separate innervation of the different sets of fibers.

Dr. Stover, of Rhode Island, then read a paper on "The Frequently Gynæcological Origin of Inherited Forms of Strumous Disease, and the Consequent Indications for Treatment," The section was shown a number of new instruments and appliances.

SECTION III. Dr. H. H. Smith then gave a demonstration, from a number of moist and dry preparations, of points referred to in his address delivered that morning. Time being granted for discussion, Dr. Gouley, of New York, objected to the use of the term "*deposition* of tubercle." He thought we did not know much about tubercle, but that, inasmuch as we are coming to regard it as a hypertrophy or hyperplasia of normal elements, the term " deposition " was incorrect; that the term " metamorphosis," if we must have such an one, would be preferable.

Dr. Sayre rose to combat the idea that hip-joint disease was largely of tubercular origin. He reported a case, which he had seen in England, of traumatic origin, where the family pedigree, as traced back for several generations, was exceptionally healthy. He alluded facetiously to the fact that in this country we kept with scrupulous care the pedigrees of our horses and cattle, but that it was very difficult to get a clear family history two generations back.

Dr. Julius F. Miner, of Buffalo, took the floor and read a paper entitled, " The Extirpation of the Thyroid Gland," illustrating the same with several specimens, which were passed around upon plates. He quoted the discouragement of the operation made by various authors, expressed the opinion that this denunciation was unwarranted, and exhibiting three specimen growths recently removed, gave their history, and the condition and experience of the patients from whom they were taken. In conclusion, Prof. Miner held that if all other means fail, some goitrous tumors may be extirpated with the knife, with reasonable hope of success.

He was asked by Dr. Sayre whether the vessels were ligated before or after division, and Dr. Miner replied that most of the blood vessels were ligated immediately after the division. Only one was generally ligated before division. Dr. Sayre then made some remarks upon the subject. He also discussed the subject demonstrated by Dr. Smith.

Dr. B. A. Watson, of Jersey City, then interested those present with an exhaustive and carefully prepared paper upon the subject, " Disease Germs; their Origin, Nature and Relation to Wounds." The conclusions drawn by Dr. Watson were:

First. That there are certain germs in the air, more particularly in the atmosphere of overcrowded hospitals, which, if permitted to enter wounds, give rise directly to living organisms, and indirectly to all septic conditions which are found as wound complications.

Second. That the successful management of wounds depends principally on the ability of the surgeon to keep the wounds under all circumstances and at all times free from germs and living organisms; and therefore the value of any method of wound treatment depends primarily on the degree of antisepsis which can be obtained by it.

Third. That the occasional discovery of a few bacteriæ in a wound, which has been treated antiseptically, does not disprove the fact that these bacteriæ arise from germs; but may be satisfactorily explained in a variety of ways, especially by the existence of germs which have not been destroyed by the means employed.

The next paper was by Dr. Hyde, of Courtland, N. Y., whose subject was "The Process of Repair in Wounds with and without Antiseptic Treatment." Dr. Hyde endorsed the use of the antiseptic treatment so far as it proved itself, though he did not regard it as being fully established at the present time. As to what treatment should be adopted, he said: "First it should aim to protect the purity of the primary exudation, that it may become as soon as possible fibro-cellular-tissue; and maintain its just relations to the sundered, injured textures, that its advancing development may not be hindered, but on the contrary, made as summary as possible.

Dr. Burns, of Philadelphia, read a brief paper on "Conservative Surgery in Compound Fractures," which embraced the history of a case which constituted, in his opinion, an entirely satisfactory example of the good results of conservative surgery under unpromising circumstances.

The Secretary read a communication from Dr. Robert Battey, of Rome, Ga., expressing regret at his inability to attend the convention.

On motion, Dr. Battey's paper on "The Permeability of the entire Alimentary Canal by Enemata, with some of its Surgical Applications," was read by title and referred to the appropriate committee.

Dr. Blodgett, of Boston, read a paper giving the history of a case of "carcinoma," with some remarks on the pathology of the disease.

Lastly, Dr. Post read a history on exsection of the articular extremities of the phalanges of the fingers and toes for the relief of deformity of those members.

SECTION IV. Transacted its first business.

Dr. Kempster read an interesting and lengthy paper on the "General Paresis of the Insane." The paper was discussed by several of the gentlemen present. Dr. Compton stated that he never saw a case of paresis among the large number of negro insane patients which he has had under his charge.

After some desultory discussion on the resolutions referred by the convention, the section adjourned.

SECTION V. The first paper read was by Dr. T. M. Stevens, of Indiana. It was composed of answers to a series of questions addressed by the Chairman of the section in a circular. In the course of his paper he made the following statements:

"A very large proportion of diseases in Indiana is due to defective drainage. It is impossible to say what the per cent. is; all forms of malarial fever are prevalent here, and as is well known they have bad drainage as a cause, even if the theory of miasm is abandoned and that of oscillation of temperature substituted; the damp moist grounds are the homes of those forms of maladies. In Indiana we might say that two kinds of general ailments are in a greater or less degree connected with so-called malaria. One form of consumption is no doubt often due to insufficient drainage. The moist and chilly air that is inseparable to locations where the surface ground is soaked with water, is favorable to the production of one form of consumption, also some forms of catarrhal affection, pneumonia and intestinal disorder; this fact is too well known to be disputed. As to diphtheria and true typhoid fever, as we think they depend upon a specific 'germ,' or some 'vital contagion,' we do not believe that moisture in the ground or elsewhere will cause them, but it may and no doubt does act deleteriously upon the system, and often places it in a condition that invites the 'specific germ' to 'such a habitat'—as for the typhoid fever, so prevalent in certain localities and seasons in Indiana, we believe that some forms of so-called typho-malarial fever often receive the more of the specific form, so nearly in many respects do they resemble each other. * * *

"The fact must be remembered that the ground may have too much water upon it to develop malaria, and if this be drained off intermittents, etc., will appear, but if drainage is continued, and the surface soil be depleted of its water of saturation, then they will disappear again. This chain of events often throws drainage into discredit; far better is it to flood the marsh than not thoroughly to remove the water of saturation."

With regard to the influence of rain-fall and drought on the purity of the water supply, he says: "There is no doubt that where there is an impervious clay soil, and in some degree with any soil, rains, somewhat in proportion to their extent and the quantity of water precipitated, contaminate the water supply as we have it here, viz., from rivers and wells, the filth and debris being washed into these at each rain—not only this, but the first stream of water is more or less impregnated with the filth, for say what we will, earth or sand is by no means a perfect filter. The country towns and cities are full of wells dug to the first stream, so that the inhabitants suffer from a double sense, viz.: the washing into each well, and impurities that find their way to the stream from which such wells derive their supply of water.

"Drought has of course only a negative effect upon the purity of water; when there is no rain to wash or percolate, such impurities are in a degree absent."

Concerning the spread of contagious disease by water contamination, he queries:

"Does the water ever usurp the place of air as a carrier of the exanthemata? Can the poison of diphtheria ever find its way into the body of another in this way? Such questions are to be answered by the sanitarian of the future. * * As the majority of the inhabitants of the country, as well as of cities, are dependent upon either wells or cisterns for their supply, if a large percentage of such are but the reservoirs for the most impure waters, it is important that knowledge of the dangers should be disseminated. But there should be a remedy suggested for every evil shown to exist, and we think there is one for the troubles we have mentioned, as efficacious as it is simple. There is no new principle involved, for it is merely a filter, nor is it new in application in this form, but it is not fully adapted, nor is its complete success acknowledged. The material to be used is

'front' or soft brick. With these a vaulted chamber is formed, resting upon the bottom of the cistern. This chamber may be from two to six barrels in capacity. The vault is arched at the top, similar in form to an old-fashioned 'bee-hive.'

"The bricks are laid in cement, but none is applied to the surface; the water flowing into the cistern filters through the soft brick into the vault, and by this means is rendered clear and sweet. The pipes from the pump enter this filter, the point of entrance being rendered tight by cement.

"Water will continually fill the vaulted chamber; each stroke of the pump partially exhausting it, will facilitate the flow, so that there is, at all times, a sufficient supply within the filter. Since we resorted to the expedient mentioned we have had no trouble whatever. * * Until some mode of filtering their water supply is adopted, the inhabitants of cities that are supplied by river water by means of water-works, know not what they drink, and may at any time suffer from the effect of impurities taken into their systems. We consider this mode of filtering water as capable of almost universal application, and it is for this reason we have spoken so fully concerning it."

Dr. Trader, of Sedalia, Mo., in his answer to the same circular, read next, made the following points:

"Notable are the epidemics of dysentery that have at several seasons since 1866, made their appearance in the vicinity of Sedalia and Georgetown. It cannot certainly be known whether the contamination was subterraneous or surface, or both combined. The springs, in the locality named, besides containing lead and antimony, may also have decayed animal and vegetable matter in solution, and it is quite probable that the latter two have a more deleterious influence to health than the former. Many families during our epidemic of dysentery, who used cistern waters, were seriously affected, especially those who had taken no precaution against contamination. Properly filtered cistern water seems so entirely free from any deleterious substances that those families using such water were comparatively exempt from the epidemic. * * Damp and overflowing cellars are, apparently, as prolific in the production of malarial disorder as are the bottom lands of our creeks and rivers. * *

In every case where cellars have been properly drained and dried, the betterment of the household was at once apparent."

The views of Dr. Carrigan, from Hempstead Co., Ark., may be gleaned from the following extracts from his reply :

" Our bowel affections, pneumonias, catarrhal affections and diphtheria, we regard as being dependent upon malaria. Consumption and typhoid fever we seldom if ever have as original complaints.

" In our prairies the soft lime rock underlies the soil at various depths. This lime rock is composed of 85 per cent. of carbonate of lime, which, of course, makes the water hard. In our sandy and timbered lands we usually get water in ferruginous clay or quicksand. In the prairies the depth of four or five hundred feet is frequently reached before getting water. In the timbered portions the wells will average about 30 feet. In the southern part of the State we have few springs. Nearly all the people use well water, which is usually freestone, clear and pleasant-tasted. Beyond doubt, from soil saturation, and from the timber curbing—which nearly all the country people use—we can account for the malarial diseases that we encounter in the high and well-drained portions of our country and in no other way.

A dry season is always a healthy season ; out of the reach of our swamps.

To show that the water in our swamps is contaminated by vegetable decay, I would state that the people who use cistern water have better health than those who use well water.

After the conclusion of this subject Dr. Jacobi, of New York, addressed the meeting in further reply to the paper read Tuesday afternoon, by Dr. Bowditch, of Boston, on the contagiousness of membranous croup and diphtheria.

Dr. Wilcox, of Hartford, Conn., asked Dr. Jacobi the cause of diptheritic diseases, and Dr. Jacobi said he could answer the question very easily by saying he did not know, but spoke about many cases and causes in his practice.

Dr. Abbott, of Hamburg, N. Y., spoke of an interesting case, where cutaneous diphtheria followed piercing of the ears, and the patient died. In less than six weeks afterward, there were fully six cases in the village, which indicated contagion.

Dr. Jacobi spoke of a case of a man who scratched a little pimple, and it was followed by erysipelas and meningitis causing death in four days afterward. He believed in a specific poison, but was not certain whether it was chemical or parasitic.

Dr. R. J. O'Sullivan, of New York, spoke of the diseases among the children in the public schools in New York. He said that within the past ten days a lady teacher had dropped dead while at her duty, and another, not being allowed to go home when ill, died three days afterward from the effects of nervous diseases incident to the imperfect health precautions in the public schools, where so many children are packed together.

Dr. Bell, of New York, then offered the following resolution, which was adopted:

*Resolved*, That Dr. E. Seguin's paper on the " Intervention of Physicians in Education " be recommended for publication in the transactions, and that Drs. F. H Hamilton, E. Seguin, R. J. O'Sullivan, of New York; Dr. D. B. Lincoln, of Boston; Dr. W. H. Van Bibbon, of Baltimore, be appointed a committee to report to the Association at its next meeting upon the practical suggestions of the said paper.

Dr. Billings, U. S. A., then exhibited the designs of the Johns Hopkins Hospital to be erected at Baltimore, and explained the improved methods of ventilation, system of caring for the sick in the ward rooms, dining room and all parts of the buildings, also the system of draining under and about the hospital, which were attentively listened to.

Dr. Jones, of Toledo, moved that Dr. Billings of the United States Army prepare a paper on the construction of hospitals, to be read at the next annual meeting of the Association, which was carried.

Papers by several absentees were read by title and referred to the Committee on Publication.

### Thursday Morning—General Session.

After the transaction of some routine and minor business, the following resolution was offered by Dr. A. N. Bell, of Garden City, and was laid over under the rules until next year:

*Resolved*, That Section IV., on Medical Jurisprudence and

Psychology, and Section V., on State Medicine and Public Hygiene, be consolidated into one section as Section IV.

Dr. N. S. Davis, Chairman of the Judicial Council, to which was referred the previous morning the matter of an amendment to the code of ethics, then reported the following:

"In obedience to the instructions of this Association, the Judicial Council, acting in the capacity of a committee, have unanimously instructed me to report to your honorable body the following amendment and addition to Paragraph 1, Article 1, of the second division of the code of ethics, under the general heading, "Of the duties of physicians to each other, and to the profession at large," and the special heading, "Duties for the support of professional characters." The same when finally adopted to be added at the end and to constitute a part of said Paragraph 1, of Article 1. The proposed addition is in these words: "and hence it is considered derogatory to the interests of the public and the honor of the profession for any physician or teacher to aid, in any way, the medical teaching or graduation of persons knowing them to be supporters and intended practitioners of some irregular and exclusive system of medicine."

It was ordered that under the rules the proposed amendment would have to be laid upon the table until the next annual meeting.

Dr. J. M. Toner, of Washington, D. C., Chairman of the Committee on Necrology, presented his annual report. It was a very voluminous document, containing sketches of seventy-five physicians who had died during the past year, and owing to its great length was not read but referred at once to the Committee on Publication.

The report of Dr. H. C. Wood, Chairman of Committee on Catalogue of National Library, being absent, his report asking for extension of time was read by the Secretary, and on his motion the request granted.

Next came the address by Dr. A. L. Loomis, chairman of Section I. He first gave a brief résume of recent advances in materia medica and physiology, making special mention of jaborandi. He then passed to the consideration of the different forms of phthisis, and especially their climatic treatment. He alluded first to the anatomical changes which occur in lung tissue in this disease, and to the pathology of the three forms, catarrhal, fibrous and tubercular.

In one class of cases the primary changes are in the cavities of the alveoli and bronchi, and are epithelial and cellular in their nature. This class would include catarrhal phthisis.

In another class of cases, the primary changes occur in the bronchial and alveolar connective tissue, and are connective tissue hyperplasias. This constitutes the variety known as fibrous.

Again, in another class of cases, the primary changes occur in the lymphoid elements of the lung, in which hyperplasia of the lymphoid elements associated with connective tissue hyperplasia form little masses or nodules, which are ordinarily termed tubercle. This class he would include under the head of *Tubercular Phthisis.* These different anatomical changes in the lungs differ so widely and give rise to such varying phenomena in the course of their development, that in order properly to estimate the value of remedial agents, the power of hygienic surroundings, and of climate to prevent or arrest their development, there must be a careful analysis of our cases, that we may determine the variety and stage of development of each case which comes under our observation.

In tubercular phthisis he had never known climate to produce favorable results, while in the other two varieties it has shown marked power in arresting and controlling the disease. There could be but little question that there were atmospheric germs which, when drawn into the lungs on inhalation, act in a chemico-local manner. They act not only upon the surface of the mucous membranes, but originate destructive processes in the lung parenchyma.

Fifteen years ago the belief prevailed that the essential climatic element for the arrest or cure of phthisis was a warm, dry atmosphere. More recent observations and investigations have settled the fact that phthisis is not necessarily hastened in its development by a low temperature, neither is it prevented or cured by a high temperature. As yet, no one has found the ideal climate for the phthisical invalid. Again, it has been claimed that the higher the altitude, the fewer were the cases of phthisis, until at a certain elevation it entirely disappeared, and that this diminution in the number of cases was due to diminished atmospheric pressure. More extended observation has demonstrated that the altitude at which this supposed immunity exists, varies with the latitude; that the nearer the approach to the equator, the higher must be the altitude in order to accomplish the desired result. This fact seems to prove that the development of phthisis does not depend upon atmospheric pressure, for the laws which govern atmospheric pressure are ever the same at a given altitude.

Dr. Schreider, in his lectures on Climatology, states that ozone and rain have the power of purifying the atmosphere, that is freeing it from organic substances, that the purifying power of ozone depends upon its oxydizing power, that while oxygen requires a considerable degree of heat before it will combine with other substances, ozone will do so at an ordinary temperature.

Ozone destroys the products of decomposition by chemically combining with them. The presence of ozone in the atmosphere is presumptive evidence that it contains no organic substances. The air of the ocean and high mountains is richer in ozone than that of the plains. As has been already said, ozone purifies the air of a locality by destroying injurious gases, and by oxydizing decomposing organic substances. It also promotes nutrition and blood changes by supplying to the respiratory organs a most active form of oxygen. Therefore, when choosing a health resort for phthisical invalids, we should give the preference to a locality in which there is constantly an excess of ozone in the atmosphere, for experience has established the fact that there the climate is especially salubrious. For some years pulmonary invalids have been recommended to take up their abode in the midst of pine forests. It has been known that they did well amid such surroundings, but "why they did well" has been an unanswered question. The more extensive and primitive the evergreen forests, the better adapted is the climate to phthisical invalids. The turpentine exhaled from these pine or hemlock forests possesses to a greater degree than any other known substance, the power of converting the oxygen of the atmosphere into ozone, thus rendering the air of these pine forests very pure, and consequently antagonistic to phthisical development. Experiment has shown that the direct inhalation of ozone has little if any power in preventing or arresting phthisical development. We must, therefore, conclude that it is not the action of the ozone upon the respiratory surfaces that renders the climate of localities where it is found in excess especially salubrious, but that by its power of destroying noxious gases and atmospheric germs the atmosphere is rendered so pure that its action is favorable upon the respiratory surfaces of those predisposed to phthisical development.

Temperature has always been regarded as of very great importance in the climatic treatment of phthisis. For a long time a warm sedative climate was regarded as the suitable one for phthisical invalids; more recently, it has been claimed that a cold climate is the favorable one, and that phthisical mortality decreases as we go northward.

An extended clinical experience will lead one to accept both views as correct to some extent.

It is not the mean temperature of a locality which is of such importance in retarding phthisical development, but it is the absence of sudden and frequent changes. Whether a cold or warm climate is indicated in any given case, can be determined only by the experience of the individual prior to the phthisical development.

The question naturally arises, is it possible to determine without a trial of the region, who shall go to the sea and who shall go the mountains. The experiments of Prof. Beneke seem to prove that tissue changes take place more rapidly on or by the sea than in the mountains; if this is the case we may readily arrive at the following conclusions: 1st. That individuals in whom the processes of tissue change do not require hastening are better in the mountains than on, or by, the sea. 2d. Persons past middle life, in whom phthisis has been developed, do better in sea than in mountain air. 3d. Phthisical invalids should not go to the mountains unless they are capable of considerable muscular activity. 4th. As a rule, phthisical individuals with an exhausted nervous system, with an overtaxed brain from excessive mental labor, or an all-absorbing occupation, yet who still retain considerable latent muscular power, will improve in the mountains, while those whose processes of tissue change require hastening or stimulating, they being in too feeble a condition to take active muscular exercise, should go to sea.

Sea air is better suited than mountain air to those who cannot bear sudden changes of temperature; while the susceptibility to such changes is greatly lessened by mountain air.

The writer's experience had led him to the following conclusions:

First—That we can expect permanent improvement in cases of developed phthisis only after a prolonged residence in the locality which experience has proved to be best suited to each individual case. Permanent favorable results cannot be obtained from an annual change of climate.

Second—That cases of *tubercular* phthisis in any stage of the disease, grow steadily and rapidly worse in all localities. Such cases do best in the quiet, well ventilated apartments of their own homes, where they can be surrounded by all those influences and circumstances which tend to make a feeble invalid comfortable.

Third. That cases of *fibrous* phthisis in every stage, whether the fibrous process commenced in the pleura or in the bronchial tubes, even after retraction of the chest walls, especially in the infra-clavicular region, is well marked, and the bronchial dilatations which accompany it, give the physical signs of extensive cavities, improve, and often reach a condition of comparative health, when they take up their residence in regions having very high altitude, such as are found in Colorado and in the Rocky Mountain range. The benefit which asthmatic and emphysematous invalids derive in these regions is most marked.

He had seen only a very limited number of cases of catarrhal phthisis permanently improved by long sea voyages or a residence in a warm climate. A large number in the early stage of this disease, going from a northern to a southern winter are temporarily improved; after the first apparently beneficial effects are passed, the degenerative inflammatory processes go on more rapidly than before.

The invalids whom he had found to be most markedly benefited by a sojourn during the winter months in a southern climate, were those convalescing from some acute pulmonary affection, in whom the delayed convalescence raises the fear of possible phthisical development, and those in whom acquired or hereditary phthisical tendencies exist, yet there may be no positive physical signs of disease of the lungs. The list of such cases is a long one, and the results obtained are most satisfactory. His favorite resorts for such cases are Aiken in South Carolina, Palatka, Enterprise and Gainsville in Florida, and Thomasville in Georgia. His best results in the stage of consolidation of the catarrhal form of phthisis have been reached in those who have made a prolonged stay (varying from one year to three years) in mountain regions with an elevation of from 1,500 to 2,000 feet. Of such regions the most positive and permanent beneficial results have been obtained in Ashville, N. C., and in the Adirondack region in New York.

His address concluded with the following practical suggestions:

"It seems to me that the necessities of our time are demanding the establishment not only of well organized and thoroughly equipped sanatariums by the sea, in the mountains, in the cold regions of the North, and in the warm regions of the South, but that our mineral springs should be utilized for the care of disease. No one doubts but they are equal if not superior to those of the old world; yet to-day we know more of the virtues of Karlsbad, Kissengen, Vichy, and Hunyadi waters, than those of Saratoga, Virginia, Arkansas, and Colorado. Has not the time come, gentlemen, when some organized action should be taken in this matter?"

Dr. White, of Buffalo, spoke in highest terms of the address,—of which a long abstract has been given on account of general interest in the subject, and then alluding to the general ignorance on the subject of ozone, offered the following resolution, which was carried:

*Resolved,* That the President appoint a committee of five members to confer with General Myers upon the subject of making observations as to the existence of ozone in various localities and take such other steps and measures in the matter as may be necessary for the success of the object.

The Committee on Nomination submitted the following list of nominees for various positions:

*President*—Theophilus Parvin, M. D., of Indiana.

*Vice-Presidents*—A. J. Fuller, M. D., Maine; W. F. Westmoreland, M. D., Georgia; John Morris, M. D., Maryland; John H. Murphy, M. D., Minnesota.

*Treasurer*—Richard J. Dunglison, M. D., Pennsylvania.

*Librarian*—William Lee, M. D., District of Columbia.

*Committee on Library*—Johnson Eliot, M. D., District of Columbia.

*Next Place of Meeting*—Atlanta, Ga.

*Time of Meeting*—First Tuesday in May, 1879.

*Assistant Secretary*—Scott Todd, M. D., Atlanta, Ga.

*Committee of Arrangements*—J. P. Logan, Chairman; H. V. M. Miller, G. G. Crawford, H. L. Wilson, J. F. Alexander, J. M. Johnson, Charles Pinckney, V. H. Talliaferro, J. T. Johnson, all of Atlanta, Ga.

*Committee on Prize Essays*—Robert Battey, Rome, Ga.; J. G. Westmoreland, Atlanta, Ga.; Wm. A. Love, Atlanta, Ga.; Robert Ridley, Atlanta, Ga.; Henry F. Campbell, Augusta, Ga.; J. H. Van Deman, Chattanooga, Tenn.

*Committee on Publication*—Dr. Wm. B. Atkinson, Chairman; Drs. T. M. Drysdale, A. Fricke, S. D. Gross, C. Wister, R. J. Dunglison, of Pennsylvania, and Wm. Lee, District of Columbia.

*Judicial Council*—To fill a vacancy caused by death, John P. Gray, Utica, New York.

In place of the seven whose terms expire at this meeting—D. A. Linthicum, Arkansas; Foster Pratt, Michigan; A. Woodward, Connecticut; J. M. Toner, District of Columbia; J. H. Van Deman, Tennessee; S. N. Benham, Pennsylvania; R. N. Todd, Indiana.

The committee also reported the following nominations for Chairmen and Secretaries of sections for 1879:

Section I, Practice of Medicine, Materia Medica and Physiology—Dr. Thomas F. Rochester, Buffalo, N. Y., Chairman; W. C. Glasgow, St. Louis, Mo., Secretary.

Section II, Obstetrics and Diseases of Women and Children—

E. S. Lewis, New Orleans, Chairman ; J. R. Chadwick, Boston, Mass., Secretary.

Section III, Surgery and Anatomy—Moses Gunn, Illinois, Chairman ; Dr. J. R. Weist, Indiana, Secretary.

Section IV, Medical Jurisprudence, Chemistry and Psychology—Dr. William M. Compton, Mississippi, Chairman ; L. M. Eastman, Maryland, Secretary.

Section V, State Medicine and Public Hygiene—Dr. John S. Billings, District of Columbia, Chairman; Dr. J. T. Reeve, Wisconsin, Secretary.

The above report was unanimously adopted.

A report, signed by Drs. Davis and Gross, concerning the recommendations in the annual address of President Bowditch, at the Chicago meeting, last year, was read by the former. It was deemed inadvisable by the committee to attempt to make the alterations in the working programme of the Association recommended by Dr. Bowditch, in that they tend to complicate rather than to simplify matters. They recommended simply the striking out of the paragraph in Section 11, of the by-laws, commencing with "Papers appropriate to the several Sections," etc., and inserting in its place the following:

"It shall be the duty of every member of the Association who proposes to present a paper or report to any one of the Sections, to either forward the paper, or a title indicative of its contents and its length, to the Chairman of the Committee of Arrangements, at least one month before the annual meeting at which the paper or report is to be read. It shall also be the duty of the President and Secretary of each Section, to communicate the same information to the Chairman of the Committee of Arrangements concerning such papers and reports as may come into their possession or knowledge for their respective Sections, the same length of time before the annual meeting. And the Committee of Arrangements shall determine the order of reading or presentation of all such papers, and announce the same in the form of a programme for the use of all members attending the annual meeting. Such programmes shall also contain the rules specified in the by-laws and ordinances concerning the consideration and disposal of all papers in the Sections."

Dr. Bowditch himself, of the same committee, submitted a minority report, maintaining his own position, at the same time admitting the cogency of argument of the majority report, and gracefully acquiescing in their judgment.

The majority report was adopted.

Next in order was the address of Dr. J. L. Cabell, Chairman of Section V. Devoting the entire address to matters pertaining directly to his Section, he gave a digest of what was doing in our own country and abroad. Considering first public hygiene, he said that the evidence of an advance in this direction with reference to the maintenance of the purity of the air within and around dwellings, consists not so much in the discovery of new facts or principles, as in more careful, exact and honest methods of sanitary engineering, in conformity with well-known laws of sanitary science. He alluded to the necessity for proper drainage and the proper action of the soil and consequent oxydation of organic matter by this means. He thought that in cities, the asphalt pavement would best preserve the atmosphere from contamination. He told how Dr. Frankland had recently investigated the conditions under which organic germs pass from sewage into air, and by means of decisive experiments had demonstrated the fact that the breaking up of minute gas bubbles resulting from fermentation or putrefaction is a cause of the suspension of solid organic particles in the air. If therefore, through the stagnation of sewage, or constructive defects which allow the retention of excrementitious matters for several days in a sewer, putrefaction sets in, then gases are generated, and the dispersion into the air of zymotic germs is very probable. It is, therefore, of the greatest importance that foul liquids should pass rapidly and freely through drainage pipes and sewers, so as to secure their discharge before putrefaction sets in.

The deleterious influence of cemeteries and sewerage in the neighborhood of a source of water supply, received due mention, as well as humidity of the soil, as a factor in the etiology of phthisis. He also alluded to the growing necessity for legislation concerning the pollution of rivers by sewage.

Taking up next the prevention and spread of contagious diseases, he spoke of the almost insuperable difficulties to be overcome in the accomplishment of the work. The epidemic character of some and the cyclical periods of others, could be seen better than they could be guarded against.

He also considered as suggestive and instructive the curious fact that those epidemics which chiefly affect certain organs of the body, are most common at the season of the year when other non-specific affections of those organs are prevalent, and in places most favorable to those diseases. Thus whooping-cough and measles generally attain their highest prevalence as epidemics in the winter and spring, when bronchitis and other affections of the air passages abound. Scarlet fever nearly always spreads most rapidly in autumn, and occasionally in the spring, and at both these seasons relaxed and ulcerated throats are most common. And so for cholera and enteric fever, cf which the maximum prevalence is in summer and autumn respectively, in accordance with an observation of Murchison, that "circumscribed epidemics, or the ordinary autumnal increase of fever, are often preceded by a great increase of diarrhœa." "It may be," as suggested by Dr. Ransom, "that the weakness of particular organs affords an opportunity for the entrance of the epidemic poison which has an elective affinity for those organs, and we may be able to meet and to ward off attacks of epidemics by paying especial attention to the prevention of diseases that weaken or predispose those organs to attack."

He then spoke of the prophylaxis of contagious disease, and mentioned what had been done with sulphurous acid, arsenic, etc. He also mentioned the difficulty of vaccinating a patient who is under the influence of arsenical preparations.

Alluding to the theory of contagium vivum, he expressed himself as opposed to the idea of spontaneous generation. The infectious matter of fevers and contagious diseases received lengthy consideration at the speaker's hands. Quoting the researches of Richardson, Burdon-Sanderson, Dalliger, Drysdale and others, he thought Prof. Tyndall had argued with pertinency and force, that "between the microscopic limit and the true molecular limit there is room for infinite permutations and combinations." He reviewed the experiments of Bastian, Tyndall, Jeffries Wyman, and Burdon-Sanderson, as well as of others.

While thus recognizing the fact that in simple septicæmia the microzymes are not the direct agents which produce the pathological results, we may justly enter a protest against the grave error of those who "maintain, in the face of all the experimental investigations relating to the subject during the last few years, that these organisms are without pathological significance. As Dr. Sanderson pertinently and justly remarks, "if these infinitely minute organisms are present in every intensely infective inflammation, we may be quite sure that they stand in important relation to the morbid process." He has further shown that bacteria grown in Pasteur's cultivated liquid, there being no putrid albuminoid matter present, are for the first crop inert, but eventually a product is obtained which possesses all the virulence of putrescent animal or vegetable infusions—a possible explanation being that the liquid becomes charged with the excretions of the bacteria or with the products of the decomposition of dead bacteria.

Now as to the doctrine of a *specific contagium vivum* for each of the specific fevers, I think it a sufficient answer to most of the objections urged against this doctrine, to remark that however plausible some of these objections may appear they are at once refuted by the conceded positive demonstration of such a contagium in a single case, namely, splenic fever. Koch having ascertained that the *bacillus anthracis* produces spores when grown in the cultivating liquid, proceeded to test the pathogenic activity of rods and spores as thus produced. The inoculation of either rods and spores into a small incision in the skin of a mouse produced splenic fever in every instance. If the tested material caused no development of rods and spores in the inoculator, it failed to produce splenic fever by inoculation. The proof is likewise nearly complete in the case of relapsing fever. It is now generally known that the discovery by Dr. Obermeier, of Berlin, of minute spiral organisms in the blood of patients suffering from relapsing fever has been fully confirmed by later observers. He then gave a brief historic sketch of the theory alluded to above, giving due meed of praise to Dr. J. K. Mitchell, of Philadelphia, for lectures delivered in 1846–47, on the cryptogamous origin of fevers.

In conclusion, Dr. Cabell thought that when we thus find innumerable analogies between the phenomena of the contagious fevers, and those connected with the development and life of certain low organisms, analogies so numerous and so close that every peculiarity in the manifestation of the fevers as to mode of development and spreading, will be found to be susceptible of interpretation in terms of the doctrine of a *contagium vivum*, and many of them not susceptible of any other explanation, and that, moreover, a positive demonstration has, it is universally conceded, been given in the case of splenic fever, not to insist upon the almost equally conclusive proof in the case of relapsing fever and diphtheria, nor upon the apparently conclusive demonstration given by Chauveau and subsequently confirmed by Sanderson and by Braidwood and Vacher, that the contagium of vaccinia and variola consists of transparent vesicles, first recognized by Beale, not exceeding, according to Sanderson, the 1-20,000 inch in diameter, it does appear that a very strong case has been made out in proof of the general doctrine in question.

Without appealing as much to what may be called popular taste, there was food for a great deal of earnest thought in this address, which was listened to with great interest by those who heard it.

Upon its conclusion, Dr. Sayre arose to a question of personal privilege, and asked that the Secretary be requested to place him upon record as opposed to the resolutions adopted at Detroit and Chicago, which declared that a fracture of all long bones could not occur without shortening. The request was granted, and the morning session ended.

### Afternoon—Work in the Sections.

Section I.—Dr. Bulkley being still absent, the subject of his paper—the rubber bandage—was discussed, especially by Dr. Martin, of Boston, who has done so much toward its introduction.

A paper on "Separation of the Ileum and Spontaneous Occlusion of the Divided Extremities," was read by Dr. Rochester, of Buffalo, who showed at the same time a valuable pathological specimen, which served as a text for his remarks.

Dr. Palmer, of Lockport, New York, exhibited a patient who had suffered from a large goitre, which he had successfully treated by subcutaneous injection of fluid extract of ergot, and read a paper on this method, which he warmly praised.

Dr. Davis then started a long discussion on the address of Dr. Loomis before the General Session. The subject of the sanitarium was discussed from various standpoints, including the extremes of favorable and unfavorable opinions. The outcome of the discussion was a resolution that Dr. Denison of Colorado be requested to study the subject of the climatic treatment of pulmonary phthisis as he found it in Colorado, and report at the next annual meeting. The section then adjourned *sine die.*

SECTION II.—A paper by Dr. Warren, of Boston, on "Connection of the Hepatic Functions with Uterine Hyperæmias, Fluxions, Congestions and Inflammations," was read by Dr. Storer, of Newport. It was a clear exposition of the dangers of treating specific diseases without due reference to general conditions.

Dr. Storer spoke strongly in advocacy of the principles put forth in the paper. The profession, he said, were in great danger of becoming too specific in the treatment of gynæcological diseases, and he felt the time had come when a retrograde movement would be a benefit.

Dr. Marcy spoke of the dangers which specialists were prone to run into in forgetting that there was anything to be watched, outside of their own territory.

Dr. John C. Irish's paper on "Dr. Burnham's Surgical Treatment of Uterine Fibroids," was read by Dr. Dunster. The subject was but briefly discussed.

Dr. E. Cutter, of Boston, was unable to read his paper on the use of electrolysis for the same disease, but made some remarks upon the subject.

A paper by Dr. Engelman, on Battey's Operation for the Extirpation of the Ovaries was read, and briefly discussed. The Section then adjourned *sine die.*

SECTION III. Dr. Hamilton, of New York, read his paper on "Exsection of the Metatarso-phalangea Ar ticulation in Valgus of the Great Toe." He alluded to various deformities, especially hallex valgus, caused mostly by ill-fitting shoes. In these cases of hallex valgus there was a subluxation of the phalanx, with almost complete obliteration of the synovial sac. In their treatment mechanical appliances were of little use. Amputation mutilates and removes an important point of support, and is more dangerous to life than excision. A subcapsular and subperiosteal excision of the metatarsal capitulum has been recommended, the toe to be brought into shape and the wound allowed to granulate. In his first case, he did not make the operation under the periosteum or capsule, but was yet successful. He now makes a flap on the inner aspect of the foot, slips a chain saw under the head of the metatarsal bone and removes it. He has been successful in a number of cases, and exhibited photographs of some of them before and after operation.

Speaking of the treatment of the incision with hot water dressings, the speaker took occasion to lay great stress upon the immense advantage of this method of treating traumatic or operative lesions.

On motion the regular order was suspended to allow Dr. Sayre to exhibit to the Section a child that had been treated and cured of Potts' disease by the application of the plaster jacket, after the method peculiar to himself. The child had taken no medicine, and was free from any prominent deformity.

Dr. Gunn offered the following resolution :

WHEREAS, This Section having expressed an opinion on long bones, and

WHEREAS, In general convention a member has asked and been accorded the privilege of recording his protesting vote, therefore,

*Resolved*, That this Section re-affirms its opinion that shortening, in cases of fractures of the long bones, is the rule in practice regardless of any of the means of treatment now in use.

The resolution was earnestly and warmly discussed by Doctors Kellar and Sayre in the negative and by Dr. Frank Hamilton in the affirmative. The resolution was finally adopted, Dr. Sayre requesting that his protest against the opinion it expressed be placed on record.

The introduction of this resolution, and its discussion, gave opportunity for display of some little personal animosity, which seemed entirely out of place, and should be deprecated by all.

Next Dr. Packard, of Philadelphia, entertained the Section with a paper on "Fractures near the Wrist Joint," which proved interesting of itself, and called forth very interesting discussion. Dr. Moore, of Rochester, claimed that in Colles' fracture there was more or less luxation of the ulna, and illustrated his remarks by quoted cases and specimens. He claimed that two weeks was long enough for such a fracture to be done up in splints, and that the tendons and soft parts would usually suffice of themselves as a splint, so that merely a sling was called for. He reduces such fractures by a method of circumduction, pulling in the direction of the fracture, and disengaging the styloid process from the annular ligament, if it shall have pierced it, as it often does. Dr. Hamilton added a few words commending and commenting upon Dr. Moore's remarks.

Dr. McGraw, of Detroit, read a paper on the "Pathology, Diagnosis and Treatment of Cancer," discussing the uncertainty of correct diagnosis before it is too late, and advocating the early removal of any tumor or induration which was not beyond all question benign. He esteemed it dangerous to leave even skin enough to cover the wound, but would have it heal by granulation, and spoke of amputating the arm and shoulder in selected cases of cancer of the breast.

Dr. Marcy, of Cambridge, Mass., contributed a paper on the use of carbolized ligatures in the operation of herniotomy. He thought too many failures in using the carbolized cat-gut ligature due to unskillful preparation. Even the slipping of knots was avoidable if the ligature were properly prepared in the manner described by Lister.

Section IV. The Section was principally occupied in hearing Dr. Deecke, special pathologist of the asylum for the insane at Utica, read a paper on "Microscopic Examinations of the Nervous Centers," which proved of much value and interest to those who listened to it. The Doctor exhibited several micro-photographs prepared by himself and Dr. Gray; and also specimen dissections mounted on glass for the microscope.

Section V. A letter from Dr. F. H. Hamilton of New York, was read declining to report at the next meeting of the Association on the subjects discussed in Dr. Seguin's paper, for the reason that he had not time to make such a report. A discussion then arose on the subject of the paper read by Prof. Cabell, the Chairman, before the general session during the morning.

On motion, Dr. R. J. O'Sullivan, of New York, was appointed Chairman, and Dr. William Clendinen, of Cincinnati, Secretary of a committee to present the report at the next annual meeting, declined by Dr. Hamilton.

A criticism of Dr. Cabell's address was then entered into by Dr. Bell, of New York, in which he made many excellent suggestions in regard to the sewerage in various parts of the country and the objectionable condition of most of the privy-vaults in villages and in fact nearly all of them outside of the larger towns and cities.

The Chairman then read a paper on "The Bearings of Hygiene on Therapeutics," by Dr. Black, of Newark, Ohio.

The writer concluded as follows:

"A pathological therapeutist is very apt to treat a chronic intermittent thus: A mercurial cathartic to clean out the abdominal viscera, then quinine in large doses, and if this does not succeed, then in larger and yet larger, to destroy or neutralize the hypothetical malaria in the system. Cases have I seen by the score, who had received such treatment for weeks and months unavailingly, who had taken quinine and iron until they were, as they expressed it, almost blind and deaf, and yet the disease persisted, with very brief cessations. As a hygienic therapeutist, and as one who has had a large experience with chronic intermittents, I unhesitatingly affirm of this latitude, that scarcely a case of intermittent fever need ever become chronic, and that even when so, proper management will hold the symptoms under control, until the tendency is wholly overcome. The outline of the method is as follows: Enquire carefully into the history of the case, and whether acclimated or not, directing special attention to each of the abdominal organs, and if much deranged administer the best active corrective at once. Then anticipate the next paroxysm with 12 grains quinine, divided into three doses, beginning its administration eighteen hours before the time of the expected chill. Repeat this amount of quinine every seventh day for four consecutive weeks, but at no other time, except when the patient's indiscretion brings on an irregular paroxysm. During the intervals administer daily, gentle remedies appropriate to correct the functions that show the most derangement. These remedies should be such as will keep the organs mainly at fault, up as near as possible, to the standard of healthy action, and no more, never allowing the secretions or excretions of any organ to sink far below the healthy standard, nor causing them to rise much above it. The diet should be strictly hygienic, and so also, of the exercise, not permitting the vital energy to be spent in toil, that should be devoted to recuperation. To guard against external variations of temperature, especially in the unacclimated, flannel should be worn next the skin. The grand condition of success lies in the method and means for keeping all the abdominal organs during every day and for several weeks, up to the standard of healthy action, and thus triumphantly raise the health above the ague-point. Quinine is invaluable for the arrest of periodicity, but nothing more."

After some discussion the Section adjourned *sine die.*

### Friday Morning—General Session.

Upon the opening of the final general session at the usual hour this morning, President Richardson announced the following ozone committee, appointed pursuant to the action taken yesterday: Dr. N. S. Davis, of Illinois, Chairman; Dr. J. S. Billings, U. S. Army; Dr. W. N. Geddings, of South Carolina; Dr. J. M. Toner, of the District of Columbia, and Dr. S. M. Bemiss, of Louisiana.

The following communication was received from the Medical Society of the State of Pennsylvania, and, after being read by the Secretary, was ordered entered on the minutes.

At the annual meeting of this body held in Pittsburgh, May, 1878, it was unanimously—

*Resolved,* That the Medical Society of the State of Pennsylvania, recognizing the advantages of the metric system, from its universality, simplicity and scientific character, does recommend the use of the same to the members of the society, and urges them to familiarize themselves with it, and to advise their students to use it exclusively when they commence their medical career.

*Resolved*, That in all communications made to this Society in which reference is made to weights and measures, the metric system only should be used.

*Resolved*, That the Secretary of this society is instructed to bring this action of this society to the notice of the American Medical Association at its next meeting, and urge upon the National Association a similar action.

The preamble and resolutions were adopted.

A resolution was offered by Dr. Seguin asking the confirmation of Drs. Sims, Drysdale and Seguin as Commissioners and Delegates for the question of medical uniformity in Europe, to report next year. Adopted.

Dr. Davis next offered the following resolution, which was adopted:

*Resolved*, That the Section on Practical Medicine, Materia Medica, and Physiology recommend the appointment by the American Medical Association of a committee of five members, to whom shall be referred so much of the recommendations in the address of the President of that Section as relates to the establishment of proper sanitaria for consumption, and the more accurate utilizing of the various mineral waters of our country, with instructions to report at the next meeting of the Association.

Accordingly, the President appointed the following members as such committee: Dr. H. I. Bowditch, of Massachusetts; Dr. A. N. Bell, of New York State; Dr. J. L. Cabell, of Virginia; Dr. S. E. Chaillé, of Louisiana; and Dr. Charles Denison, of Colorado.

The President announced the following delegates:

*To European Medical Societies*—Drs. Sims, Drysdale, Seguin, Daly, Halberstadt, Levis and W. H. Pancoast.

*To the Canadian Medical Association*—Drs. Brodie, Todd, E. N. Brush and W. Clarke.

The following resolutions concerning certain legal relations of the insane, offered by Dr. Foster Pratt, of Michigan, in Section V, and referred to the Association, were received and adopted:

*Resolved*, That the personal restraint of the insane is an essential element of the medical treatment of their disease, the use of which, as a therapeutical agency, may be justified by their insanity, just as the use of it as a public agency, for the prevention of injury to person or property, is justified by their dangerous conduct.

*Resolved*, That while none question the necessity for specific statutory provisions to regulate the restraint of those insane persons who are wholly or partly a public charge; we maintain,

That it is the duty of relations and friends, and it is also their natural and inherent right, whether declared or understood by statute, to restrain and to care for their sick or insane relations as private patients at their expense, in their home, or in a legally recognized and regulated hospital; and

That the exercise, by them, of so much restraint as is essential to their proper treatment of his disease, is not a violation of his rights of personal liberty; and

That their duty and right to exercise such remedial restraint are subject to State surveilance or legal limitations, only so far as may be necessary to prevent their neglect of that duty or to punish their abuse of the right.

The following report of the committee to secure the appointment of State Boards of Health was presented and adopted:

Your Committee, consisting of the President and Permanent Secretary, who are required to report annually the results of their efforts for the organization of State Boards of Health, respectfully report that they have addressed the Governor of each State, where a Board of Health has not been organized, a memorial. A few Executives have courteously acknowledged this communication and expressed their earnest desire to further our efforts. We are happy to announce that three additional State Boards have been organized, making nineteen in all, viz.: Alabama, California, Colorado, Connecticut, Georgia, Illinois, Kentucky, Louisiana, Massachusetts, Maryland, Michigan, Minnesota, Mississippi, New Jersey, North Carolina, Tennessee, Rhode Island, Virginia, Wisconsin.

Dr. X. C. Scott called up the resolution of last year, creating a new Section of Ophthalmology, Otology and Laryngology, to be known as Section VI, and moved its adoption. Carried.

On motion of Dr. E. Smith, of Detroit, Dr. H. Knapp, of New York, was made Chairman of the new Section, and X. C. Scott, of Ohio, was made Secretary.

The address of the Chairman of Section IV, Dr. Kempster, was then called for. This was principally composed of a consideration of "The Relations of Pathology to the Motor Centers." The speaker first gave a sketch of the use of the microscope in studying the histology of mental disease, and a review of the histological changes which have been discovered in such cases. Allusions to the researches of Fritsch, Hitzig and Ferrier were frequently made. He discussed the relations of the meninges to the convolutions, and quoted Crichton Browne's studies upon adhesions of the membranes over cortical motor centers as a cause of epilepsy and transient hemiplegia.

He alluded to cases of reported cure of general paresis of the insane, and considered them merely such remissions as do occur in the course of this disease. In support of the theory of localization of motor centers, he adduced numerous instances, a few of his own, and some drawn from the writings of Charcot, Dreschfeld, Ferrier and others. He mentioned the discovery of degenerative changes in the centers for members which had been amputated. In speaking of the views recently put forward by Brown-Séquard, on this subject, he said the weight of testimony and authority was greatly against him. When considering the researches of others upon the sensitiveness of the dura-mater, he described two cases in his own experience where convulsions had been caused by grasping with forceps the dura, which had been exposed by accident.

He had but brief space to devote to medical jurisprudence, but mentioned a recent Wisconsin case where the knowledge test was not considered sufficient proof of insanity. He hoped the time would come when experts would be called by the *court* when it should be deemed expedient.

The address was received with close attention.

The Secretary next read the annual report of the Treasurer, Dr. R. J. Dunglison, of Philadelphia. The report concluded as follows:

"The Treasurer is in constant receipt of applications from members for volumes of the transactions of the Association which are entirely out of print. As some of the members are in possession of scarce volumes, which they may not wish to retain, the Treasurer will willingly act as a medium of exchange, and thus accommodate those who are anxious to complete their sets." [Here followed a financial statement.]

The following report of the committee on Prize Essays was read by the local Secretary and adopted:

"Your committee to determine the merits of prize essays would respectfully report: That they have had three separate papers submitted to their inspection. Two of these papers present subjects of very great interest and show original researches, but are too imperfect in the estimation of the committee to command a prize. The remaining paper, in the judgment of your committee, is fully up to the requirements. Indeed the paper is so elaborate as to fill a large space in the volume of the transactions of the Association. The paper should be considered as *two* and not as *one*. The analysis of 789 cases of operation on the carotid artery, and the careful and minute measurements of the artery and its branches in 121 subjects, showing the range of variation and the per centage of the same, followed by inferences, bold and original, naturally constitute a paper complete in itself. Another one on the same plan with reference to the innominate and subclavian, being an analysis of 300 cases, and the observation of 52 subjects, is presented to us in such a manner that we may consider the whole as one prize, or they may compete for both.

"Your committee believe that both prizes should be awarded to the two essays by one person."

On opening the sealed envelope the name of the successful essayist was found to be Dr. John A. Wyeth, of New York city.

Dr. J. J. Caldwell, of Maryland, offered a resolution creating a new Section upon "Neurology and Electrology." Action was deferred for one year.

Dr. Maddox, of Baltimore, Md., offered another resolution creating a Section on "Diseases of the Genito-Urinary Organs, including Dermatology and Syphilis." Laid over.

Appropriate resolutions in relation to the life and death of

Prof. Henry, of the Smithsonian Institute, were offered by Dr. Toner and adopted.

Dr. Parvin, the President elect, was then introduced by President Richardson, with a few complimentary remarks, and the former returned his thanks for the honor conferred in eloquent and fitting terms.

Dr. Van Deman then offered a series of resolutions embodying the thanks of the Association, to the committees of arrangement, the officers, the people of Buffalo, and the ladies.

The Association then adjourned to meet at Atlanta, Ga., next year.

The number of delegates registered was five hundred and fifty. A pleasant feature of the meeting, to the writer at all events, was the comparatively large number of the younger class of practitioners chosen to represent the interests of the profession.

The receptions tendered by citizens of Buffalo were elegant in the extreme, and the social festivities ended with an excursion to Niagara Falls Friday afternoon.

It may justly be asked what amount of scientific work was accomplished by so large a gathering? The writer feels compelled to say that the amount of strictly original work exhibited was not amazing in either extent or importance. But it must, in candor, be said that most of the papers presented either admirable résumés of their subjects, or presentations of facts in a condensed and accessible shape. No one could have been present without feeling that he had received an equivalent for the trouble or expense of the trip.

A most admirable and important feature of the meeting, to which we have endeavored to do justice in this report, was the position given to subjects and discussion pertaining to matters of State Medicine, and public hygiene. The address of Dr. Cabell, the paper of Dr. Seguin, and hints and allusions in many of the papers or reports read, furnish ample evidence that the Association is doing a noble work in this direction, which it should be the object of every member to foster and encourage.

In conclusion, the writer acknowledges his obligations to the Buffalo daily press. Had its daily reports been accessible to the readers of this journal, there had been no call for this summary.

### Report of a Lecture on the Morphology of the Blood in Syphilis.—By Dr. E. Cutter, of Boston.

This lecture was delivered as an extra treat during one of the evenings of the Association meeting: and while not purporting to be classed among its proceedings, and although other attractions of a social nature were offered, yet the interest it excited, and the audience it drew, certainly make a short report of it very desirable. It was profusely illustrated on the screen by the stereopticon, with photographs of the doctor's own preparations.

He first spoke of the morphology of fungi and algae, and their distinctive differences, which were that fungi give off carbonic dioxide at night, and contain neither starch nor chlorophyl, while algae give off oxygen in the day time, and contain both starch and chlorophyl. He then spoke of, and briefly described spores and sporidia; and, alluding to the number of known species, said that there were fourteen thousand species of algae and eight thousand species of fungi, known to and described by naturalists. All this was introductory to speaking of the presence of fungi in the blood.

He alluded to Salisbury's and Lostorfer's researches, and spoke modestly of his own patient and laborious investigations in this subject, extending over a period of several years.

Speaking then of the connection of fungi with syphilis, he said he had dissected with extreme care, more than a hundred chancres, and invariably found fungous filaments beneath them. The authors alluded to above had found that in this disease, the white corpuscles were enlarged, and the blood contained bacteria and mycelial filaments. He showed photographs of the yeast cell and of different fungi at the same time, with the sporidiæ or bacteriæ, regarded by some as separate organizations, by others as true spores. He then showed a variety of healthy red and white blood corpuscles, some enlarged so that the image of one corpuscle was sufficient to occupy the whole area of a large screen. He next showed syphilitic blood whose white corpuscles were much larger, and which also contained mycelial threads and bacteria, and one white cell whose walls seemed to have ruptured and extruded these spores—a phenomenon which the Doctor said he had seen occur.

The object of the lecture was to exhibit and insist upon the following characteristics displayed by syphilitic blood—the increased size of the white corpuscles, and the presence of bacteria-like spores and mycelium-like filaments, which are distinctly and always of a *coppery color*. Similar changes are noted in the blood of consumptives, and of patients suffering from erysipelas, diphtheria and other diseases, but while the fungi may present other colors, they never have the coppery hue which those above described possess. He thought the time might come when we could detect in this way the so called pre-tubercular stage of phthisis. In order to see these fungi, one must take the microscope to the bedside of the patient, and examine the blood as soon as collected. He thought this was one reason why they had been so seldom seen.

Dr. Cutter is one of the *very* few men who can handle a 1-75th inch objective; many of his photographs having been taken with a 1-50th and 1-75th. His apparatus, of which he exhibited a picture, is simple. His modesty was evidently a characteristic of the man—he claimed to be more a willing witness than a discoverer.

